# Perceptions of trained laypersons in end-of-life or advance care planning conversations: a qualitative meta-synthesis

**DOI:** 10.1186/s12904-018-0354-9

**Published:** 2018-08-06

**Authors:** Elizabeth Somes, Joanna Dukes, Adreanne Brungardt, Sarah Jordan, Kristen DeSanto, Christine D. Jones, Urvi Jhaveri Sanghvi, Khadijah Breathett, Jacqueline Jones, Hillary D. Lum

**Affiliations:** 1 Internal Medicine Residency, University of Colorado School of Medicine, University of Colorado Anschutz Medical Campus, Aurora, CO USA; 2 University of Colorado Skaggs School of Pharmacy and Pharmaceutical Sciences, University of Colorado Anschutz Medical Campus, Aurora, CO USA; 3 VA Eastern Colorado Geriatric Research Education and Clinical Center, Denver, CO USA; 4 Division of Geriatric Medicine, University of Colorado School of Medicine, University of Colorado Anschutz Medical Campus, 12631 E. 17th Ave, Mail Stop B179, Aurora, CO 80045 USA; 5 Health Sciences Library, University of Colorado Anschutz Medical Campus, Aurora, CO USA; 6 Division of Hospital Medicine, University of Colorado School of Medicine, Anschutz Medical Campus, Aurora, CO USA; 7 College of Nursing, University of Colorado Anschutz Medical Campus, Aurora, CO USA; 8 Division of Cardiovascular Medicine, Sarver Heart Center, University of Arizona, Tucson, AZ USA

**Keywords:** Volunteers, Communication, Hospice care, Palliative care, Terminal care, Lay health navigators, Advance care planning, Peer educators

## Abstract

**Background:**

Laypersons including volunteers, community health navigators, or peer educators provide important support to individuals with serious illnesses in community or healthcare settings. The experiences of laypersons in communication with seriously ill peers is unknown.

**Methods:**

We performed an ENTREQ-guided qualitative meta-synthesis. We conducted a systematic search of MEDLINE, PsycINFO, CINAHL, Cochrane Library, and AMED to include qualitative studies with data regarding communication and laypersons in advance care planning, palliative care, or end-of-life settings. Study quality was appraised using a standardized tool. The analysis identified key domains and associated themes relating specifically to laypersons’ perspectives on communication.

**Results:**

Of 877 articles, nine studies provided layperson quotations related to layperson-to-peer communication associated with advance care planning (*n* = 4) or end-of-life conversations (*n* = 5). The studies were conducted in United Kingdom (n = 4) or United States settings (n = 5). The synthesis of layperson perspectives yielded five main domains: 1) layperson-to-peer communication, focusing on the experience of talking with peers, 2) layperson-to-peer interpersonal interactions, focusing on the entire interaction between the layperson and peers, excluding communication-related issues, 3) personal impact on the layperson, 4) layperson contributions, and 5) layperson training. Laypersons described using specific communication skills including the ability to build rapport, discuss sensitive issues, listen and allow silence, and respond to emotions.

**Conclusions:**

Published studies described experiences of trained laypersons in conversations with peers related to advance care planning or end-of-life situations. Based on these layperson perspectives related to communication, programs should next evaluate the potential impact of laypersons in meaningful conversations.

## Background

Advance care planning is a process that supports adults at any age or stage of health in understanding and sharing their values, goals, and preferences regarding future medical care [1]. Advance care planning is associated with increased hospice use, decreased hospital admissions, reduced medical care costs, and increased patient satisfaction [2, 3]. To increase participation in advance care planning, national recommendations suggest raising public awareness of advance care planning and enabling people to think about future medical care planning in their own life situations [4–6]. One strategy to increase community engagement and promote advance care planning is through non-medical laypersons such as volunteers, community health navigators, and peer educators [7, 8].

Trained laypersons have been involved in supporting advance care planning among general older adult populations and specific populations such as ethnic minorities with multiple comorbidities, patients with end-stage renal disease, and patients with cancer [9–11]. These layperson-based programs suggest that individuals value the opportunity to exchange stories with peers who belong to their community group, are a similar age, or share similar experiences [12]. The involvement of volunteers in advance care planning conversations is a natural extension of the long-standing role volunteers have played in interdisciplinary hospice and palliative care teams [13–15].

Laypersons in hospice and palliative care settings serve in multiple capacities, including providing physical, spiritual, and emotional comfort to patients and family caregivers; assisting with information exchange and referral support (e.g., acting as a “bridge to the hospice”); socialization; and companionship [16–19]. Layperson-to-peer communication related to living with serious illness occurs in multiple settings including hospitals and clinics, palliative care programs, hospice programs, and community settings [7, 11, 20–22]. Given the formal and informal involvement of laypersons in communicating with individuals with serious illness, the specific experiences that non-medical laypersons have related to end-of-life communication, including advance care planning conversations, warrants close examination.

Systematic reviews that summarize layperson perspectives on communication related to end-of-life situations are lacking. To address this gap, we performed a meta-synthesis of qualitative studies to address the study question: “What are the perspectives of laypersons on communication with individuals with serious illness or advance care planning?” The intention of this study is to provide a rich description of how trained non-medical laypersons engage in layperson-to-peer conversations related to advance care planning or end of life situations, including palliative care and hospice care.

## Methods

### Design

This study is an interpretive thematic synthesis which uses a structured team-based meta-synthesis approach consistent with the ENTREQ standards. Specifically, we extracted salient information about each study, developed descriptive data-driven themes, and then synthesized themes through a process called reciprocal translation [23, 24]. We conducted a comprehensive search to identify articles on non-medical laypersons (i.e., volunteers, patient navigators, peer educators) in communication related to serious illness or advance care planning. We use the term “layperson” to streamline presentation of the results, while acknowledging differences in how various non-medical trained laypersons may be compensated, trained, and integrated into community or healthcare-based programs. We use the term “peer” in recognition that some of individuals that laypersons interacted with were in community-based settings and could be considered a peer, even if they did not personally know them. We also use the term “patient” in recognition that some individuals were in a healthcare context. We chose to perform a meta-synthesis because it provides a mechanism for exploring layperson-to-peer communication across a variety of settings from multiple studies. As a rigorous systematic interpretive study of a defined body of qualitative research, this process produces new knowledge beyond the individual studies and does not include quantitative studies. The analysis involves an integrative synthesis with the following assumptions: 1) the whole published study, not just participant quotations, is treated as qualitative data for interpretation; 2) a multidisciplinary analytic team adds context variation to study interpretation, and 3) when qualitative studies include similar findings, they can be amassed to draw larger and different interpretative meaning [23, 24].

### Search strategy and study selection

A comprehensive search was performed by a medical librarian (K.D.) on March 20, 2017. Table 1 summarizes the key search terms used. Relevant publications were identified by searching the following databases: MEDLINE, PsycINFO, CINAHL, Cochrane Library, and AMED. No limiters were used for language or publication date. Publication/source types were limited in PsycINFO and CINAHL to exclude dissertations, theses, and book chapters to improve efficiency of searching and to ensure all included studies had been peer-reviewed and were easily discoverable. Appendix 1 describes the comprehensive search strategies for each database. Reference lists of included studies were hand-searched for additional relevant studies.

**Table 1:** Search strategy and study selection

Search terms	1. Volunteers OR lay navigators OR peer groups 2. Advance care planning OR advance directives OR palliative care OR hospice 3. Education OR experience OR sharing OR encouraging 4. Qualitative methods OR phenomenological study OR focus groups OR grounded theory OR observation
Inclusion criteria	1. Qualitative methods 2. Participants are non-medical peers (i.e., volunteer, patient navigator, peer educator) 3. Setting related to advance care planning, palliative care, hospice, or end-of-life
Exclusion criteria	1. Non-English language 2. Not full papers (i.e., abstracts, posters) 3. No extractable data from peers 4. No data relating to communication

The inclusion and exclusion criteria are presented in Table 1. One author (H.L./J.D./A.B.) examined titles for general relevance to the study question of layperson perspectives on communication with individuals with serious illness or advance care planning. One author (J.D./A.B.) examined study abstracts for relevance, and then two authors (H.L. and J.D.) independently reviewed full studies based on the inclusion and exclusion criteria. The final inclusion of nine studies in the meta-synthesis was confirmed by the study team.

### Quality appraisal

The quality appraisal is an important first step in a meta-synthesis and is a process of immersion into the data. It provides a deeper understanding of each article and helps the team determine the relevance and value of each study toward understanding key findings of the meta-synthesis. To assess study quality (Appendix 2), all articles were independently reviewed using the McMaster University tool [25] by at least two members of an multidisciplinary team including a nurse researcher (U.S.), a palliative care-trained geriatrician (H.L.), and two hospital-based physician researchers (C.J. and K.B.). The tool assesses for the presence or absence of 17 quality domains, including additional subdomains, for a total of 22 items that together address study rigor and other qualitative methodological issues. Any appraisal differences were resolved by consensus and input from another team member (S.J.) who has expertise in qualitative methods. To aid in comparing study quality, each domain received 0 points for No, 1 point for Yes. Not applicable (N/A) ratings were excluded from the total possible score. Scores for each domain were summed and divided by the total possible score (22 minus number of “N/A”) multiplied by 100 to provide an overall quality score with a possible range of 0 to 100%. The appraisal was not used to exclude articles.

### Meta-synthesis

Using a meta-synthesis approach based on Thomas and Harden, [24] we extracted study aim, design, methods, type of layperson participants, and main findings of the original studies. Three authors (H.L., J.D., A.B.) reviewed all articles, extracted layperson quotations, and coded meaningful ideas within and across studies. We used an inductive approach for thematic analysis to identify themes and analyze similarities and differences across the studies [24]. In studies with mixed methods, the analysis focused on the qualitative portion of the study. The process was iterative, building consensus through visual mapping of broader domains, themes, and subthemes; naming and renaming; and contextualizing themes through team discussion and re-immersion into the articles to determine whether the emerging results resonated with the original data. Congruent with a meta-synthesis approach, we then used a reciprocal translation approach to create a reciprocal theme table that displayed the synthesized domains and themes alongside themes from the original studies [24]. We maintained an audit trail of decisions and presented and received feedback from multidisciplinary palliative care researchers and clinicians on the derived themes and primary data to contextualize our findings and maintain a high degree of rigor.

## Results

**Fig. 1:**
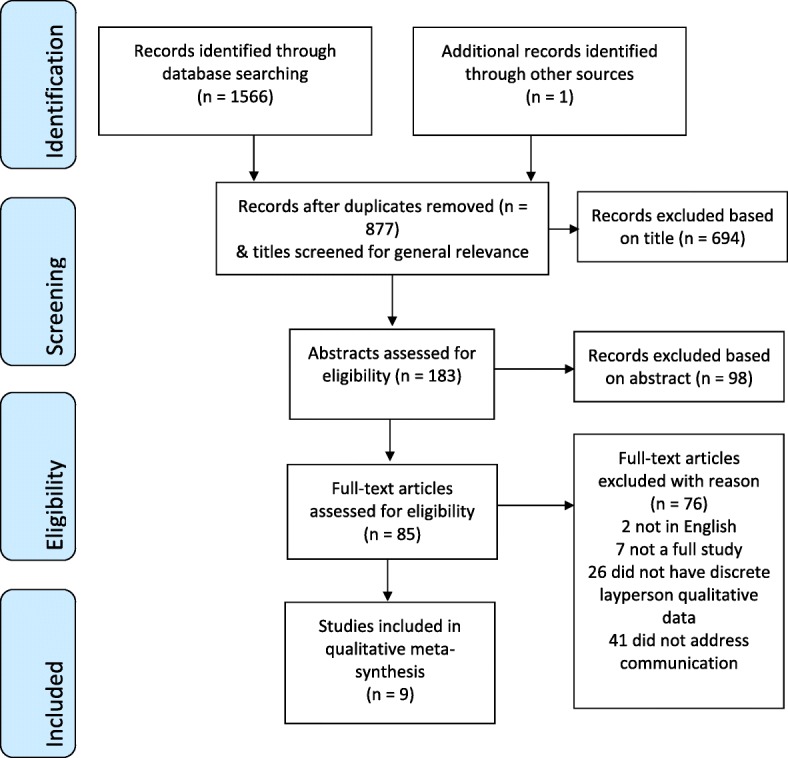
PRISMA flow diagram

Among 1566 titles identified with the initial search strategy, 690 were duplicates. One additional study was found by hand searching. Of 877 titles screened for general relevance to the study question, 694 titles were removed. Next, 183 abstracts were screened based on the inclusion criteria, and an additional 98 were removed. The full text of 85 articles were assessed, and 76 were excluded (two were not in English, seven were not full studies, 26 did not have discrete qualitative layperson data, and 41 did not address communication). Nine studies remained eligible for inclusion in the meta-synthesis as shown in the PRISMA diagram (Fig. 1) [26].

Table 2 shows study characteristics. Studies were conducted in the United Kingdom (UK) or the United States (US) and published between 2002 and 2017. Most studies used a qualitative descriptive approach with interviews, focus groups, or a combination. Two studies included a participatory action approach and one study used ethnography. Four studies specifically focused on communication related to advance care planning; whereas, five studies addressed the role of laypersons in communicating with patients who were hospitalized at the end-of-life, had palliative care needs, or were receiving hospice care. Studies included laypersons as volunteers, peer educators, or lay health navigators. Sample sizes ranged from 8 to 351 participants. The combined qualitative data from the nine studies represent a total of 692 laypersons.

**Table 2:** Studies of layperson perspectives on layperson-to-peer communication related to serious illness or advance care planning

Study	Study aim	Design and method	Participants and setting	Results from primary study	Overall score from critical appraisal of study quality
1 Berry and Planalp, USA, 2009 [30]	To explore ethical issues hospice volunteers confront in their work	Thematic analysis of interviews	39 hospice volunteers in urban and rural areas in Southwestern US. Mean age 64, 76% female, 100% White	Four themes of ethical issues: 1) dilemmas about gifts, 2) patient care and family concerns, 3) issues related to volunteer roles and boundaries, and 4) suicide/hastening death.	60%
2 Brighton et al., UK, 2017 [20]	To explore hospital volunteers’ end-of-life care training needs and learning preferences, and the acceptability of training evaluation methods	Thematic analysis of focus groups	25 hospital volunteers with at least 3 months experience. Mean age 50 (range 19–80 years), 76% female, highly ethnically diverse sample	Four themes emerged: 1) preparation for volunteering role; 2) end-of-life care training needs, including a) communication skills, b) understanding grief and bereavement, c) understanding spiritual diversity, d) understanding symptoms at end-of-life, and e) volunteers’ self-care; 3) learning preferences, including a) teaching methods, b) teachers, c) optional vs mandatory training, d) consolidating learning; and 4) evaluation preferences.	95%
3 Clarke et al., UK, 2009 [36]	To evaluate whether researchers successfully worked with peer educators to develop and pilot an education program for advance care planning	Participatory action research; analysis of questionnaires, field notes, interviews	5 “older adult” peer educators from community organizations, who were research advisors and volunteer peer educators	Peer educator findings from the program development process included: 1) enjoying project meetings, 2) involvement in reviewing material, 3) enhanced awareness of advance care planning, 4) training encouraging action, and 5) training enabling action.	61%
4 Foster, USA, 2002 [28]	To describe volunteer-patient relationships and communication at the end-of-life	Narrative ethnography using interviews, observations, small groups	9 hospice volunteers and researcher over 12-months in 1 hospice	Three themes related to volunteer-patient relationships emerged: 1) focus on the life of the patient – “The patient is alive”, 2) volunteer prioritizes the patient – “It’s not about me”, and 3) importance of presence – “Being there”.	60%
5 Jones et al., UK, 2015 [29]	To evaluate volunteers’ experiences of advance care planning in a hospice	Mixed-method descriptive case studies, data from open-ended questions	10 advance care planning -trained hospice volunteers completed questionnaires, providing 23 statements for analysis	The first theme was benefits of being an advance care planning volunteer, including a) positive interactions, b) gratitude shown by peers, and c) personal impact. The second theme was challenges of being an advance care planning volunteer, including: a) no engagement by peer, b) negative attitude of caregiver, c) being asked for inappropriate advice, and d) denial by peers.	50%
6 Planalp and Trost, USA, 2008 [22]	To understand difficult communication issues or dilemmas experienced by hospice volunteers, patients, and their families	Qualitative analysis of data from 3 open-ended questions	351 hospice volunteers from urban and rural areas in Southwestern US. Mean age 55 (range 15–88 years), 75% female.	Three themes of communication issues were identified: 1) denial between the dying person and their caregiver/family; 2) dealing with many negative feelings experienced by the patient and their caregiver/family; 3) family issues, including a) within-family conflicts, b) conflicts about patient care/treatment, c) financial/estate issues, d) unresolved relationship issues. Sources of communication difficulties were physical and/or mental impairments that made it difficult to talk with the dying person.	68%
7 Planalp et al., USA, 2011 [27]	To describe conversations volunteers had with patients that they considered meaningful	Qualitative analysis of data from open-ended questions and in-depth interviews	350 hospice volunteers from 32 hospices in Southwestern US completed questionnaires, 31 volunteers interviewed	Prominent themes about meaningful conversations were: 1) meaning of life, experiences and life stories, 2) talk about death and spirituality, 3) families and relationships, 4) shared interests with volunteers, 5) unfinished business, and 6) loss of capacities. Volunteers appreciated gaining life lessons.	60%
8 Rocque et al., USA, 2017 [11]	To evaluate implementation of lay navigator-led advance care planning	Mixed-methods design, including thematic content analysis of lay navigator interviews	26 lay navigators in Respecting Choices advance care planning Facilitator training in Southeastern US. Mean age 45, 81% female; 39% Black, 58% White.	Navigators identified key facilitators and barriers of implementation of advance care planning. Facilitators included physician buy-in, patient readiness, and prior advance care planning experience. Barriers included space limitations, identifying the “right” time to start conversations, and personal discomfort discussing end-of-life.	95%
9 Seymour et al., UK, 2013 [37]	To report volunteers’ perspectives on a advance care planning peer education program and feelings on role of volunteer peer educator	Participatory action research; mixed methods including interviews and focus groups	24 older adult volunteers and 8 care staff. 25% below 55 years, 9% over 75 years; 81% white, 6% black	At 6 and 12 months after training, the volunteers’ perspectives related to 1) personal and emotional implications of being a peer educator, and 2) report of community engagement activities in the year after peer education training.	84%

In the initial immersion into the data and assessment of study quality, study quality varied with overall quality scores ranging from 50 to 95% (Appendix 2). Across the nine studies, areas of poor quality were description of sampling methods; description of study site; identification of researchers’ biases; and confirmability of data to minimize bias.

### Meta-synthesis of themes

**Fig. 2:**
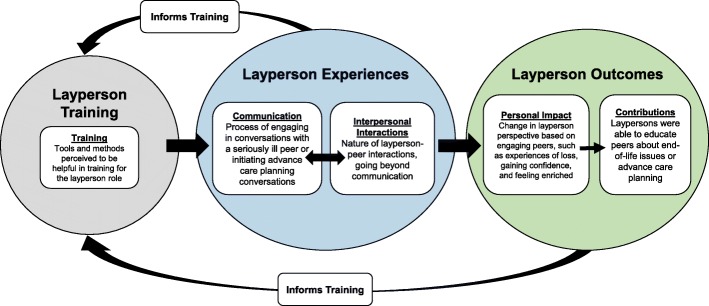
Layperson perspectives on layperson-to-Peer communication related to training, experiences, and outcomes

Across nine studies, five major domains with themes and subthemes emerged related to laypersons’ involvement in communication related to end-of-life or advance care planning conversations. The major domains were 1) *layperson-to-peer communication*, focusing on the experience of talking with peers, 2) *layperson-to-peer interpersonal interactions*, focusing on the entire interaction between the layperson and peers, excluding communication-related issues, 3) *personal impact on the layperson*, 4) *layperson contributions*, and 5) *layperson training*. Figure 2 provides a graphical representation of the domains and associated themes. Table 3 presents each domain, related themes, and subthemes, as well as themes from the original studies to provide additional context.

**Table 3:** Domains and themes exploring layperson perspectives about communication in serious illness or advance care planning

Derived domains, themes and subthemes	Theme from primary study (# refers to Table 2)	Exemplar layperson quotations for each subtheme (# refers to Table 2)
Domain 1. Layperson-to-peer communication		
Building rapport (Subthemes: Building trust; Developing relationships over time)	Benefits as an advance care planning: Positive interactions (5); Difficult communication: Volunteer’s role (6); Navigator-level facilitators to advance care planning (8)	Of course, all of these patients have a relationship with me. Have rapport with me. I have seen them several times previously before I bring it up, so they know who I am and what I look like. They trust me. (8) I’ll explain the type of services I can offer. The first time I may just mention it and tell them that one of the things that we can offer is advance care planning and we can talk about that another time if you’re more interested. (8)
Talking about sensitive issues (Subthemes: Advance care planning; Prognosis; Death; Family/caregiver issues; Bereavement; Suicide)	Patient care and family concerns (1); Suicide and Hastening Death (1); End-of-Life Care Training Needs: Symptoms; Communication skills (2); Training encouraging action (3); The Patient is Alive (4); Personal Impact (5); Difficult communication: Denial; Dealing with pain/discomfort; Negative feelings of caregiver/family (6); Meaningful conversations: Life stories; death and spirituality; families and relationships; unfinished business (7); Patient-level barriers to advance care planning (8); Increased confidence in the topic (9)	Before [advance care planning] always felt like a major topic, you know ‘Oh how am I going to raise this?’ … [the training has] made it seem like something more natural to talk about, not to feel so awkward about discussing end-of-life matters and decisions … so that was really helpful. (9) It was just…had I known how serious the prognosis was, I would have handled the situation much differently. So, you know, it is very difficult going in cold sometimes because you can end up really putting your foot in your mouth. (2) It means that you have the opportunity to say to a family member who is quite distressed, ‘Actually, it is okay. This is all that’s happening’, and that can be the most powerful thing you can possibly say, to know that actually that’s okay. It is not anything out of the ordinary. (2, discussing dying) The most meaningful conversation was with the mother and the young kids. The topic was her concerns and fears about her family. (7) The most difficult thing seems to be business-type problems that need to be dealt with prior to or after the death. Families seem to find it an insensitive topic but usually recognize the necessity. (6) She was so miserable, she wanted me to contact this Dr. Kevorkian, and so trying to talk her out of it without disparaging her wishes, or you know, disappointing her…she wanted to be right with God, but she was talking about having, you know, ending her own life. It was her discussion, and I didn’t promote it or anything. It was just a discussion to talk about, so I guess talking about that with somebody would be considered...kind of like I shouldn’t be doing it, but it interested me to talk to her about it, and she was a very open person. (1)
Listening and allowing silence	End-of-Life Care Training Needs: Communication skills (2); Being There (4); Shared interests (5); Difficult communication: Physical/mental impairment (6); Meaningful conversations: Life Stories (7)	Most striking was how much he needed somebody to listen and not judge. (7)
Responding to patient and family emotions	End-of-Life Care Training Needs: Communication skills (2); Difficult communication: Negative feelings (6)	[The patient] was in one of the rooms where you have to wear extra protective clothing as well, and I remember his children there. And they were really upset, and obviously me and him were having…He is telling me about times he went to Jamaica, and we’re talking, but I could see that his children were visibly upset. So I asked them if they wanted me to leave, and they were like no, no, no. To them, they are seeing their dad, someone who has looked after them, in an ill position, but I didn’t really know what to say to them. (2)
Communication facilitators (Subthemes: Physician endorsement; Healthcare team involvement)	System-level facilitators to advance care planning (8)	I want to get into the clinics and I want to have the doctor say to the patient, ‘Your navigator is gonna come in, and they’re gonna cover all of this, including advance directive, and I think that everybody should get it done.’ (8)
Communication barriers (Subthemes: Physical or cognitive limitations; Health literacy limitations; Limited time for advance care planning conversations; Cultural norms)	End-of-Life Care Training Needs: Communication Skills (2); It’s Not About Me (4); Personal Impact (5); Difficult communication: Physical/mental impairment; Religious differences (6); Patient-level barriers to advance care planning (8); System-level barriers and facilitators to advance care planning (8)	I think the hardest issue for me was when my lady couldn’t think of the word she wanted to say. She would get very frustrated and would cry. The best way I could get through this was to try to understand the concept of what she was talking about, then redirect the conversations. Sometimes I would use humor or we would listen to her favorite music. (6) Sure, they’re anxious about whether or not they want to be on what they deem to be life-supportive equipment or whether they want a CPR. It kinda gets into the medical jargon. (8) I found it stressful with the pressure of completing an advance care planning quickly. It was like hitting a target. (5) It’s just hard to deliver given our time with the patient and actually space to actually have a private conversation with them. (8) There’s a cultural suspicion about it … they call people who would discuss these things like death panels…I don’t know if that’s just our region or whatever…the difficulty is, I think, getting people to understand this is for their benefit. (8)
Domain 2: Layperson-to-peer interpersonal interactions		
Discomfort with peer’s situation (Subthemes: Witnessing symptoms; Witnessing family distress)	Patient care and family concerns (1); End-of-Life Care Training Needs: Symptoms; communication skills (2); Being There (4); Difficult communication: Denial; Dealing with pain and discomfort; Family issues (6)	I actually thought he was dead because he didn’t say anything for a while, and I was like ‘Oh no, what am I going to do’. Because he had really heaving breathing, I don’t know what was actually wrong with him. Air would get caught in him and it seemed like he was choking, so I told the nurse straight away. And she was like, ‘No, don’t worry.’ (2) The family wouldn’t mention that the patient is dying in front of him and refused to discuss it. They were really in denial. I told them that he knows he is dying - they were shocked that I knew because the patient told me. This opened up dialogue between them. We all felt some relief. (6)
Uncertainty of layperson role (Subtheme: Responding to patient/family requests; Gifts; Responding to symptoms)	Patient care and family concerns (1); Dilemmas about Gifts (1); Volunteer roles and boundaries (1); Difficult communication: Conflicts about patient care (6)	The person asked me if I would…If I would stay 8 h - for the day, and I told ‘em, “Now, that’s really not my job.” I said, “It’s a four-hour shift and I’ll be glad to do that, but 8 h…that’s really a lot.” (1) …and I said, “I really can’t accept that.” She says, “You will hurt me if you don’t accept that.” So I thought, “What do I do? What do I do? It’s almost like a gift.” And I know it didn’t cost her anything, but that’s not the principle, and I said, “Do I give it away?” And she was very adamant that I accept it, and so I didn’t want to hurt her feelings, so I took it, right or wrong or indifferent… (1)
Interpersonal differences between layperson and peer (Subthemes: Cultural/religious differences; Socioeconomic differences)	End-of-Life Care Training Needs: Understanding spiritual diversity (2); The Patient is Alive (4); Difficult communication: Religious differences (6)	The one that stood out for me the most is understanding spiritual, cultural, and environmental aspects of dying because nowadays we are living in a society where people are very religious. And even though there may be some patients that aren’t religious, you just have to respect what somebody believes. (2)
Domain 3: Personal impact on the layperson		
Building meaningful relationships (Subthemes: Learning from the peer; Receiving gratitude; Experiencing loss)	End-of-Life Care Training Needs: Self-care (2); The patient is alive; Being There (4); Benefits as an advance care planning volunteer: positive interactions; gratitude shown by peers; personal impact (5); Peer loss (6); Meaningful conversations: Shared interests; Gaining life lessons (7); Implications of being a peer educator (9)	Some things I don’t understand and he explains to me. He wants to be informed about the future. He’s not - he doesn’t stop living here in the present. He makes me understand that, too, the way he talks about politics. He’s all into that. I’m ashamed sometimes, I think, “Gosh, you know, I’m 23 and I don’t know that a bomb exploded here or there.” I think he keeps his life, very simple, day-to-day, to live in the moment. Maybe that’s a lesson I’m supposed to learn. (4) The benefits are the gratitude for engaging with people to discuss such delicate issues at this very sensitive time in their lives. (5)
Gaining awareness of end-of-life (Subthemes: Personal reflection; Personal application; Personal difficulty with mortality)	Training encouraging action; training enhancing awareness (3); It’s Not About Me (4); Difficult communication: Denial (6); Navigator-level barriers to advance care planning (8); Implications of being a peer educator (9)	The subject of dying and death is very delicate and how it is presented makes a difference. Over the months, the training we received has helped me become comfortable with the subject and, to a certain extent, more knowledgeable. (3) It certainly brought home to me that I myself might find this situation occurring in my life and yet I’d not done anything about it. So it made me think a lot and it made me talk to my family about it. (3) The difficulty is just dealing with somewhat your own - not issues, but your own reserves about this. (8)
Domain 4: Layperson contributions		
Educating others	Training encouraging action (3); Training enabling action (3);	I certainly didn’t feel fazed by any of the questions that we were asked and, fortunately, other people participating in the group also answered questions, it wasn’t just left to us as the leaders. (3) Older people have always been good educators for younger people but with support we can educate each other on more serious issues like these. (3)
Engaging in community outreach	Community engagement activities (9)	I’ll be quite frank, I certainly wouldn’t have got as involved as I am if I hadn’t done it (the training). After the last training day we had a meeting [and] said well okay, we’ll go and meet these people and see what they’re doing ... one thing we found was who knows what is out there as far as care provision. So we went and saw the hospice. We went and saw the PCT [Primary Health Care Trust]. We went and saw the Council. ... We literally pressed our PCT to get an (end-of-life) care strategy together because we went and saw them and we said okay we’re going to hold an information day and we will invite them to come and talk. So it pressurized them into having something ready. (9)
Domain 5: Layperson training		
Strategies (Subthemes: Experiential learning; Meetings over time; Supervision; Group-based learning)	Learning preferences (2); Program development process; Training encouraging action (3); Helpful additional supervision (5); Personal and emotional implications of being a peer educator (9)	If I know that it actually happens and this was the scenario that someone actually faced, it makes it seem more than just an exercise [sounds of agreement]. (2) I think role play is probably one of the best ways. So do I, because it puts you on the spot. (2) We’ve had a chance to take things in and think about it and then come to another meeting and that’s been helpful, I think. (3) Actual structured supervision with feedback. (5)
Instructional personnel	Learning preferences (2); Training encouraging action (3)	I think both [staff and volunteers], because you will get the professional experience or the professional knowledge of the situation and they might have been dealing with it for years, so their training, and then a bit from the volunteers because they are hands on also. So I think both is important. (2) There were three groups. In each, there were 6–8 people from various backgrounds, and then two peer educators supported by a researcher. We rotated roles within the group, taking turns to be a facilitator, co-facilitator and observer. (3)
Materials	Learning preferences (2); Reviewing training materials (3); Suggestions for programme improvement (5)	The material is too detailed for use in a peer education guide. Most people, myself included, would find it most daunting to prepare an advance directive after reading the book. The subject presentation is too complicated for a peer guide where simplicity should be of the essence. (3) General information about what is available locally so we can signpost more effectively. (5)

#### I. Layperson-to-peer communication

The domain of layperson-to-peer communication includes six key themes: a) *building rapport*, b) *talking about sensitive issues*, c) *listening and allowing silence*, d) *responding to patient and family emotions*, e) *communication facilitators*, and f) *communication barriers*. These themes describe the layperson’s process of engaging in conversations with a seriously ill peer or initiating advance care planning conversations.

Laypersons focused on *building rapport*, which included building trust and developing relationships over time. They noted that longitudinal relationships over multiple encounters allowed for time and space to have unhurried discussions about sensitive subject matter. One volunteer described the process as follows,

“You just … need to hit the ball back over the net when you’re talking to someone… who has that disorder. You’re not seeking things, you’re not negotiating a peace treaty here, and you’re not making a business deal here... All you need to do is just hit the ball back over the net. They’re gonna hit it right back to you, you just hit it back” [27].

Moreover, laypersons noted that being perceived as a “peer” enhanced trust; “I think she sees me as a friend, also someone to maybe pass on some of her wisdom” [28]. Laypersons often felt most comfortable focusing on life-related subject matter through “life review” conversations.

Layperson-to-peer communication involved *talking about sensitive issues*, such as advance care planning, prognosis, death, family and caregiver issues, bereavement, and suicide. Laypersons were able to engage in these diverse conversation topics because of their training and by overcoming their own obstacles, such as initial avoidance of death-related subjects and lacking sufficient knowledge of a peer’s medical or social situation. When laypersons did initiate difficult conversations with a peer, it was ultimately met with a sense of relief. Through discussions with laypersons, hospice patients and loved ones often overcame denial of death and were able to address end-of-life practicalities, such as funeral planning and care of pets.

*Listening and allowing silence* was another theme of layperson communication. Some laypersons indicated that silence was anxiety-provoking because initially they worried about their contribution to the conversation and how the patient would perceive them. They reported that over time, they relinquished their self-concern and focused on the patient. They learned that listening, and being present or “in the moment,” were the greatest gifts they could give because patients often needed someone to listen without judgement.

*Responding to patient and family emotions* was another theme of layperson-to-peer communication. Laypersons perceived several negative emotions experienced by patients including fear, anger, regret, guilt, loss of dignity, and feeling like a burden to their families. Laypersons also described that families appeared to experience grief, fatigue, discouragement, feeling trapped, and feeling guilty for wanting the process to be over. Families were fearful of losing their loved one, fearful of not being present at the time of death, and concerned about the loved one’s pain and not being able to alleviate it. Laypersons wanted to learn how to sensitively and appropriately respond to these emotions.

Laypersons identified *communication facilitators* of layperson-to-peer communication, including physician endorsement and healthcare team involvement. For example, they felt that physician endorsement of layperson-led advance care planning conversations would help to reinforce its importance. They also felt that the support and involvement of healthcare team members helped when a patient’s questions surpassed the layperson’s role. One volunteer noted,

“If they start askin’ questions that I’m not sure of, then I’ll get a nurse. I’ll ask her questions, and I’ll come back to ‘em. I’ve had one that would ask about, well, how long would they keep feeding me before they would turn me off or whatever...I wasn’t sure, so I went and got an MD to answer the question for me” [11].

Laypersons also identified several *communication barriers*. Laypersons noted peer-related obstacles to conversations, including denial of death, lack of readiness, limited health literacy, and family conflicts. Specific barriers included physical or cognitive impairments, such as Parkinson’s disease or dementia, or the active dying process. In these situations, non-verbal communication became even more important when a patient’s disease made verbal communication difficult. Patients seemed to be reassured by the layperson’s presence. Specific to advance care planning conversations, laypersons noted health literacy limitations, including how the peers they were supporting seemed overwhelmed by medical information and jargon provided by the healthcare teams. At the broader community or healthcare system-level, a lack of time and space for advance care planning conversations, lack of widespread healthcare provider support, and cultural suspicion about talking about death and dying were the primary communication barriers. A volunteer in an advance care planning program stated, “I found it stressful with the pressure of completing an advance care planning quickly. It was like hitting a target” [29].

#### II. Layperson-to-peer interpersonal interactions

The second domain describes the nature of the layperson-to-peer interactions, going beyond communication. The themes included: a) *discomfort with the peer’s situation*, b) *uncertainty of the layperson role*, and c) *interpersonal differences between layperson and peer*. *Discomfort with the peer’s situation* encompassed witnessing symptoms and witnessing family distress. Laypersons described feeling helpless when observing patients’ symptoms and not knowing what to do. Some were upset when witnessing a patient’s distress over not being able to communicate, and felt unsure of how to help a patient with dementia. Witnessing a patient’s or family’s denial about death, hurtful family interactions, or emotional distress also caused discomfort. Laypersons identified these circumstances as opportunities for further training and desired clear preparation for encounters with distress, the dying process, and death.

A second theme that characterized interpersonal interactions was laypersons’ *uncertainty of their role*. This theme included uncertainty regarding responding to patient/family requests, gifts, and responding to symptoms. A commonly cited reason for this uncertainty was the position of being neither friend nor provider. Laypersons felt that they were in a nebulous in-between role. One volunteer described:

“[The patient] was in pain, and made it very clear that he wanted his morphine, which is an absolute…you know, no-no. I’m not supposed to be dispensing medication. It was, for me, a very uncomfortable and difficult situation to be in, ‘cause on the one hand, you don’t want to watch a human being suffer. On the other hand, it was made very clear to me that, you know, ‘this is something you don’t do!’” [30].

Laypersons recounted requests from patients and families that were inappropriate for this in-between role, such as dispensing pain medications, staying at the facility beyond their volunteer shift, or performing conspiratorial favors, such as throwing away an item that the patient didn’t want his family to see. This nebulous role also meant receiving gifts put them in an awkward position. If the layperson was a personal friend, they would have no problem receiving gifts. If they were a healthcare team member, they would have clear boundaries for declining gifts. Laypersons’ uncertainty also related to responding to symptoms or the peer’s self-care needs. They were uncertain about their role when advocating for the patient when concerns about a patient’s care or needs were raised. Laypersons often needed to navigate their role with the peer, family members, and healthcare team members, each of whom may have had different expectations of the layperson’s role and appropriate level of involvement.

*Interpersonal differences between layperson and peer* was a third theme and included the subthemes of cultural or religious differences and socioeconomic differences. Some laypersons perceived that religious differences could be a barrier. For example, some laypersons described feeling disconnected from a peer whose beliefs contradicted their own, while others admitted it was difficult to refrain from sharing their own beliefs. Awareness of these differences, however, did not necessarily cause a rift between the layperson and peer. One layperson recalled a patient with whom she connected despite their very different socioeconomic backgrounds:

“We instantly connected because we left out all the bullshit and just connected on a human level. And there’s a lot of female connection that we have, too. We connect as two women. We can talk about men, our husbands, what society expects of us as women, and what we want out of life. So she, I realized, shares the dreams and desires and aspirations that I have. We’re sisters under the skin” [28].

#### III. Personal impact on the layperson

The third domain is the personal impact on the layperson as they engaged in training, meeting peers with serious illness, or initiating advance care planning conversations. A key theme of this domain was *building meaningful relationships*, including learning from the peer, receiving gratitude, and experiencing loss. Laypersons describe “enriching” and “rewarding” experiences, gleaning wisdom from their patient as a “living history.” They felt rewarded by gratitude from the patient, which they felt accounted for the challenges of discussing death and dying. By forming strong connections with patients, however, they also experienced loss. Laypersons commented on the difficulty of letting go of friendships that had formed, stating:

“It does affect you at times when you know someone, you may be seeing them… [and] during two or three weeks you get to know them, and then they are gone” [20].

Personal impact on the layperson also included *gaining awareness of end-of-life*. Laypersons elaborated on this awareness in subthemes of personal reflection, personal application, and personal difficulty with mortality. Several laypersons commented that death became less daunting as a result of their experiences, especially when seen through the eyes of a peer with a positive outlook. They gained a better appreciation of how others approach end-of-life issues, and a deeper understanding of loss. They applied these lessons to their own lives, feeling better prepared to support those who had lost someone and how to advocate for their own wishes. One layperson commented that his experience had been a “re-education,” and he had become more compassionate as a result. Not all experiences were positive. Some laypersons discussed their own anxieties about mortality limited their ability to help patients.

#### IV. Layperson contributions

The fourth domain is layperson contributions and includes the themes of *educating others* and *engaging in community outreach*. Through effective training programs, laypersons discovered that they were able to educate their peers about end-of-life issues or advance care planning. They felt satisfaction when seeing the results of their hard work, such as completing a workbook for advance care planning with a peer. Some felt emboldened to engage their own families in end-of-life care discussions and even expanded their work into the wider community. For example, some trained peer educators hosted information sessions and meetings with local community stakeholders. They became further involved in local and national organizations aimed at increasing awareness of death and advance care planning.

#### V. Layperson training

The final domain is layperson training. Several studies described the processes of preparing laypersons to be peer navigators, educators, or hospice or hospital-based volunteers to support individuals with serious illnesses or to initiate advance care planning conversations. Layperson training focused on communication skills and provided laypersons with knowledge, experience, and confidence to address specific communication issues, as well as the broader role of supporting a peer. In addition to the content-focused suggestions that laypersons had related to the aforementioned domains, layperson input specific to training included *strategies*, *instructional personnel*, and *materials*. Suggestions for training strategies included experiential learning, meetings over time, supervision, and group-based learning. Laypersons preferred experiential learning, using real case examples and role playing, over computer-based “e-learning” or virtual classrooms. In terms of timing, they valued attending trainings that continued after starting the layperson role because they were able to learn from their real-life experiences, reflect between sessions, and receive on-going support from other laypersons. Structured supervision with feedback was another training need. Lastly, laypersons felt co-leading a group discussion related to advance care planning, rather than independently leading groups, helped peer education to go more smoothly.

A second theme related to layperson training was *instructional personnel*. Laypersons found that the most effective training was provided by experienced lay volunteers and healthcare professionals (e.g., palliative care providers), in addition to the program coordinators. The third training suggestion related to *materials*. Laypersons felt that simpler, more layperson-friendly materials were more effective than advance care planning printed materials that used complicated jargon. They also suggested that advance care planning materials for peers be based on stories or examples to make the concepts more understandable.

## Discussion

### Main findings of the study

This meta-synthesis addresses the study question: “What are the perspectives of laypersons on communication with individuals with serious illness or advance care planning?” We provide an integrated synthesis of the thoughts and experiences of non-medical laypersons as they communicate with peers experiencing serious illnesses, end-of-life care, or related to advance care planning conversations. In focusing on layperson-to-peer communication, this analysis describes commonalities in how trained laypersons approached and experienced conversations. It also highlights the variety of interactions, social or clinical context, benefits, and challenges of those conversations. The findings provide additional support to the role of laypersons in having meaningful conversations, though healthcare provider or physician endorsement of the layperson role may improve their effectiveness [8, 14]. Together with specific input from laypersons on their training needs, these findings can inform best practices for training and ongoing support systems for community or healthcare system-based programs that involve lay individuals. The synthesized results provide a foundation for the design and adaptation of peer-based programs that focus on communication skills and training.

This study offers insight into the benefits and challenges of laypersons’ engagement in advance care planning conversations. While other research studies focus on advance directive documentation, this study describes how engaging with seriously ill patients or peers in advance care planning conversations can be a challenging yet rewarding experience from the layperson’s perspective [31]. A future analysis should also include perspectives of the peer/patient and family caregivers [32]. Laypersons also shared similar sentiments regarding end-of-life communication: being with the patient and his/her loved ones and talking about death could be anxiety-provoking or uncomfortable, especially with inadequate training. Many laypersons and patients still found the experience to be positive. Laypersons specifically described increased awareness of end-of-life issues for themselves and, in turn, initiated conversations with families, friends, and sometimes their broader community. Thus, the investment of training a layperson for involvement in palliative care, hospice, or other programs to support seriously ill individuals may yield community-level benefits related to discussions about death and dying. Additionally, for programs that utilize older adult volunteers, this analysis aligns with a theoretical benefit between volunteering and successful aging through opportunities for communication [33, 34].

As a meta-synthesis, this study included individual studies that involved laypersons in highly varied settings, including hospices, hospitals, and community-based outreach programs to enhance advance care planning conversations. There were diverse types of non-health laypersons, including hospital volunteers, hospice volunteers, lay health navigators, and peer educators. The laypersons may have been part of a specific communication-based program or may have had opportunities for communication as part of their broader role. Although this meta-synthesis provides access to context variation within and across studies, the application of the key findings must be re-contextualized to the particular type of layperson, type of peer/patient, and program implementation setting. The laypersons’ suggestions on training, planning for program sustainability, and legal and ethical aspects of the involvement of laypersons in communication-based roles need to be adapted to regional or national policy considerations. The unique position of laypersons may require training measures specific to them because laypersons experienced uncertainty in their role, being neither a caregiver nor a health care professional. However, laypersons reported building and using communication skills such as building rapport, responding to patient and family emotions, and talking about sensitive issues which are skills also used by health care professionals. Because these skills are commonly used by health and social care professionals, there may be opportunities to adapt existing training models for use in layperson programs. Additionally, further study could evaluate the potential benefit and challenges of shared training, at least in part, for health care providers and laypersons in communication skills for a particular program. Given the significant difference in the role of a healthcare provider and a trained layperson, skills which may seem transferable between the two may still require different training methods and would require further evaluation.

Further research on the impact of laypersons in advance care planning or end-of-life conversations is warranted. This meta-synthesis focuses on the perspectives of laypersons, but future work should focus on the perspectives of patients, family members, and members of the healthcare team regarding the role and impact of laypersons. Prior to widespread adoption of laypersons in this role, specific evaluation of the safety and potential effectiveness of trained laypersons on communication and other meaningful person-centered outcomes is needed.

### Limitations of the study

This study has several limitations. As a meta-synthesis, we did not have access to the original data sets, including complete transcripts or field notes, and were limited in our ability to interpret the linguistic and cultural context of the published quotations. Additionally, the focus on qualitative studies, inclusion criteria, and exclusion criteria resulted in unintentionally limiting the geographical location of the studies to the US and the UK. For example, in choosing to exclude grey literature such as dissertations, theses, and book chapters, we may have biased the findings toward established programs that had desire and ability to publish in peer-reviewed journals. The literature search yielded studies involving laypersons in palliative care or end-of-life settings conducted elsewhere, such as Canada and Uganda, but those studies did not specifically examine communication or use qualitative methods [18, 35]. Still, the majority of studies relating to volunteer or other layperson experiences are based in the US, Canada, or UK, making it difficult to broadly apply the findings of this meta-synthesis beyond these regions. Future work should include grey literature as this literature may have insights from additional settings, making the results of a meta-synthesis more broadly applicable. Additionally, the scope of this study did not include quantitative outcomes related to advance care planning programs involving trained laypersons [31]. An additional limitation to the study is that there was no layperson on the research team contributing to the analysis of themes.

## Conclusions

The findings from layperson perspectives on communication with peers experiencing serious illness or related to advance care planning have practical implications. Since volunteers are more likely to commit to an activity that is personally satisfying, volunteer laypersons may constitute a reliable and cost-effective way to enhance advance care planning efforts and support individuals with palliative care needs, especially in community-based settings [35]. Training, and even paying, laypersons could be a viable alternative to training existing healthcare providers in specific advance care planning communication skills, especially in resource-limited settings. Moreover, because laypersons may have more time or common life factors on which to establish rapport, laypersons are uniquely positioned to engage in end-of-life conversations with peers experiencing serious illnesses.

In conclusion, we synthesized the perspectives of a diverse group of laypersons who were involved in communicating with individuals with serious illnesses or as part of advance care planning programs. Together the studies described the involvement of laypersons in meaningful conversations with their peers and outlined interpersonal interactions, personal impact, contributions, and training that laypersons experienced. Laypersons may complement and potentially enhance the work of healthcare providers in meeting the educational and psychosocial needs of individuals and their family caregivers in palliative care settings. Programs that involve laypersons should include training specifically for layperson-to-peer conversations related to the end-of-life period, as well as a mechanism for providing ongoing support to maximize and sustain the impact of the layperson’s role.

## Appendix 1

**Table 4:** Search strategies. Comprehensive search strategies used to identify articles for each database

MEDLINE, including Ovid MEDLINE Epub Ahead of Print, In-Process & Other Non-Indexed Citations, and Ovid MEDLINE Daily (1946-present)	(exp Peer Group/ or exp. Volunteers/ or exp. Mentors/ or exp. Patient Navigation/ or (voluntary or peer* or volunteer* or mentor* or navigator* or lay*).tw,kf.) and (exp Advance Care Planning/ or exp. Hospices/ or exp. Hospice and Palliative Care Nursing/ or exp. Palliative Medicine/ or exp. Palliative Care/ or exp. Terminal Care/ or (Living Will* or Medical Power* Attorney or Health Care Power* Attorney or Healthcare Power* Attorney or advance* care plan* or advance* directive* or advance* health care plan* or advance* healthcare plan* or advance* medical plan* or hospice* or palliative or terminal care or end-of-life).tw,kf.) and (exp Patient Education as Topic/ or exp. Counseling/or exp. Information Dissemination/ or exp. Teaching/ or exp. Consumer Health Information/ or (discussion* or coaching or navigation* or navigating or learning or awareness or training or conversation* or engag* or promotion* or story or stories or experienc* or sharing or educator* or education* or educating or encourag* or teaching or counseling or information or knowledge or communicat*).tw,kf.) and (exp Qualitative Research/ or exp. Grounded Theory/ or exp. Interviews as Topic/ or exp. Focus Groups/ or exp. Nursing Methodology Research/ or exp. anecdotes as topic/ or exp. narration/ or exp. “surveys and questionnaires”/ or exp. personal narratives as topic/ or exp. Observational Studies as Topic/ or exp. interview/ or exp. personal narratives/ or exp. observational study/ or (qualitative or ethnograph* or phenomenol* or grounded theor* or purposive sampl* or hermeneutic* or heuristic* or semiotics or lived experience* or narrat* or life experience* or cluster sample* or action research or observational method* or content analys* or thematic analys* or constant comparative method* or field stud* or theoretical sampl* or discourse analys* or focus group* or ethnological research or ethnomethodolog* or interview* or mixed method* or mixed model* or mixed design* or survey* or questionnaire* or anecdote*).tw,kf.)	Results = 745
PsycINFO via Ovid (1806 to March Week 2 2017)	(exp peers/ or exp. volunteers/ or mentor/ or (voluntary or peer* or volunteer* or mentor* or navigator* or lay*).ab,ti.) and (exp advance directives/ or exp. Palliative Care/ or exp. hospice/ or (Living Will* or Medical Power* Attorney or Health Care Power* Attorney or Healthcare Power* Attorney or advance* care plan* or advance* directive* or advance* health care plan* or advance* healthcare plan* or advance* medical plan* or hospice* or palliative or terminal care or end-of-life).ab,ti.) and (exp peer education/ or exp. Community Counseling/ or exp. Educational Counseling/ or exp. Peer Counseling/ or exp. information dissemination/ or exp. teaching/ or exp. consumer education/ or exp. death education/ or (discussion* or coaching or navigation* or navigating or learning or awareness or training or conversation* or engag* or promotion* or story or stories or experienc* or sharing or educator* or education* or educating or encourag* or teaching or counseling or information or knowledge or communicat*).ab,ti.) and (exp qualitative research/ or exp. grounded theory/ or exp. Interviews/ or exp. Narratives/ or exp. surveys/ or exp. questionnaires/ or exp. narratives/ or (qualitative or ethnograph* or phenomenol* or grounded theor* or purposive sampl* or hermeneutic* or heuristic* or semiotics or lived experience* or narrat* or life experience* or cluster sample* or action research or observational method* or content analys* or thematic analys* or constant comparative method* or field stud* or theoretical sampl* or discourse analys* or focus group* or ethnological research or ethnomethodolog* or interview* or mixed method* or mixed model* or mixed design* or survey* or questionnaire* or anecdote*).ab,ti.) *Publication types were limited to (“0100 journal” or “0110 peer-reviewed journal” or “0120 non-peer-reviewed journal” or “0130 peer-reviewed status unknown”).*	Results = 277
CINAHL Cumulative Index to Nursing and Allied Health Literature (CINAHL) via EBSCOhost	(MH “Peer Group” OR MH “Volunteer Workers” OR MH “Volunteer Experiences” OR TI (voluntary or peer* or volunteer* or mentor* or navigator* or lay*) OR AB (voluntary or peer* or volunteer* or mentor* or navigator* or lay*)) AND (MH “Advance Care Planning” OR MH “Advance Directives+” OR MH “Hospice Patients” OR MH “Hospice and Palliative Nursing” OR MH “Hospices” OR MH “Terminal Care+” OR MH “Palliative Care” OR TI (Living Will* or Medical Power* Attorney or Health Care Power* Attorney or Healthcare Power* Attorney or advance* care plan* or advance* directive* or advance* health care plan* or advance* healthcare plan* or advance* medical plan* or hospice* or palliative or terminal care or end-of-life) OR AB (Living Will* or Medical Power* Attorney or Health Care Power* Attorney or Healthcare Power* Attorney or advance* care plan* or advance* directive* or advance* health care plan* or advance* healthcare plan* or advance* medical plan* or hospice* or palliative or terminal care or end-of-life)) AND (MH “Patient Education+” OR MH “Counseling+” OR MH “Teaching” OR MH “Death Education” OR MH “Consumer Health Information+” OR TI (discussion* or coaching or navigation* or navigating or learning or awareness or training or conversation* or engag* or promotion* or story or stories or experienc* or sharing or educator* or education* or educating or encourag* or teaching or counseling or information or knowledge or communicat*) OR AB (discussion* or coaching or navigation* or navigating or learning or awareness or training or conversation* or engag* or promotion* or story or stories or experienc* or sharing or educator* or education* or educating or encourag* or teaching or counseling or information or knowledge or communicat*)) AND (MH “Qualitative Studies+” OR MH “Ethnological Research” OR MH “Action Research” OR MH “Phenomenological Research” OR MH “Ethnographic Research” OR MH “Field Studies” OR MH “Grounded Theory” OR MH “Multimethod Studies” OR MH “Survey Research” OR MH “Phenomenology” OR MH “Focus Groups” OR MH “Interviews+” OR MH “Narratives” OR MH “Surveys+” OR MH “Observational Methods+” OR MH “Discourse Analysis” OR MH “Thematic Analysis” OR MH “Content Analysis” OR MH “Qualitative Validity+” OR TI (qualitative or ethnograph* or phenomenol* or grounded theor* or purposive sampl* or hermeneutic* or heuristic* or semiotics or lived experience* or narrat* or life experience* or cluster sample* or action research or observational method* or content analys* or thematic analys* or constant comparative method* or field stud* or theoretical sampl* or discourse analys* or focus group* or ethnological research or ethnomethodolog* or interview* or mixed method* or mixed model* or mixed design* or survey* or questionnaire* or anecdote*) OR AB (qualitative or ethnograph* or phenomenol* or grounded theor* or purposive sampl* or hermeneutic* or heuristic* or semiotics or lived experience* or narrat* or life experience* or cluster sample* or action research or observational method* or content analys* or thematic analys* or constant comparative method* or field stud* or theoretical sampl* or discourse analys* or focus group* or ethnological research or ethnomethodolog* or interview* or mixed method* or mixed model* or mixed design* or survey* or questionnaire* or anecdote*)) *Dissertation/thesis was excluded as a source type.*	Results = 359
Cochrane Library (via Wiley, including Cochrane Database of Systematic Reviews, Database of Abstracts of Reviews of Effect, Cochrane Central Register of Controlled Trials, Cochrane Methodology Register, Health Technology Assessment Database, and NHS Economic Evaluation Database)	#1 → MeSH descriptor: [Peer Group] explode all trees #2 → MeSH descriptor: [Volunteers] explode all trees #3 → MeSH descriptor: [Mentors] explode all trees #4 → MeSH descriptor: [Patient Navigation] explode all trees #5 → voluntary or peer or volunteer or mentor or navigator or lay:ti orab orkw (Word variations have been searched) #6 → MeSH descriptor: [Advance Care Planning] explode all trees #7 → MeSH descriptor: [Hospices] explode all trees #8 → MeSH descriptor: [Hospice and Palliative Care Nursing] explode all trees #9 → MeSH descriptor: [Palliative Medicine] explode all trees #10 → MeSH descriptor: [Palliative Care] explode all trees #11 → MeSH descriptor: [Terminal Care] explode all trees #12 → “Living Will” or “Medical Power of Attorney” or “Health Care Power Attorney” or “Healthcare Power of Attorney” or “advance care planning” or “advance directive” or “advance health care plan” or “advance healthcare plan” or “advance medical plan” or hospice or palliative or “terminal care” or “end of life”:ti orab orkw (Word variations have been searched) #13 → MeSH descriptor: [Patient Education as Topic] explode all trees #14 → MeSH descriptor: [Counseling] explode all trees #15 → MeSH descriptor: [Information Dissemination] explode all trees #16 → MeSH descriptor: [Teaching] explode all trees #17 → MeSH descriptor: [Consumer Health Information] explode all trees #18 → discussion or coaching or navigation or navigating or learning or awareness or training or conversation or engagement or promotion or story or stories or experience or sharing or educator or education or educating or encouragement or teaching or counseling or information or knowledge or communication:ti orab orkw (Word variations have been searched) #19 → MeSH descriptor: [Qualitative Research] explode all trees #20 → MeSH descriptor: [Grounded Theory] explode all trees #21 → MeSH descriptor: [Interviews as Topic] explode all trees #22 → MeSH descriptor: [Focus Groups] explode all trees #23 → MeSH descriptor: [Nursing Methodology Research] explode all trees #24 → MeSH descriptor: [Anecdotes as Topic] explode all trees #25 → MeSH descriptor: [Narration] explode all trees #26 → MeSH descriptor: [Surveys and Questionnaires] explode all trees #27 → MeSH descriptor: [Personal Narratives as Topic] explode all trees #28 → MeSH descriptor: [Observational Studies as Topic] explode all trees #29 → MeSH descriptor: [Interview] explode all trees #30 → MeSH descriptor: [Personal Narratives] explode all trees #31 → MeSH descriptor: [Observational Study] explode all trees #32 → qualitative or ethnography or phenomenoly or “grounded theory” or “purposive sample” or hermeneutics or heuristics or semiotics or “lived experience” or narrative or “life experience” or “cluster sample” or “action research” or “observational method” or “content analysis” or “thematic analysis” or “constant comparative method” or “field study” or “theoretical sample” or “discourse analysis” or “focus group” or “ethnological research” or ethnomethodology or interview or “mixed method” or “mixed model” or “mixed design” or survey or questionnaire or anecdote:ti,ab,kw (Word variations have been searched) #33 → (#1 or #2 or #3 or #4 or #5) and (#6 or #7 or #8 or #9 or #10 or #11 or #12) and (#13 or #14 or #15 or #16 or #17 or #18) and (#19 or #20 or #21 or #22 or #23 or #24 or #25 or #26 or #27 or #28 or #29 or #30 or #31 or #32)	Results = 37
Allied and Complementary Medicine (AMED) via Ovid (1985 to March 2017)	(exp Peer group/ or exp. Voluntary workers/ or exp. Mentors/ or (voluntary or peer* or volunteer* or mentor* or navigator* or lay*).ab,ti.) AND (exp advance directives/ or exp. hospices/ or exp. palliative care/ or exp. terminal care/ or (Living Will* or Medical Power* Attorney or Health Care Power* Attorney or Healthcare Power* Attorney or advance* care plan* or advance* directive* or advance* health care plan* or advance* healthcare plan* or advance* medical plan* or hospice* or palliative or terminal care or end-of-life).ab,ti.) AND (exp patient education/ or exp. counseling/ or exp. teaching/ or (discussion* or coaching or navigation* or navigating or learning or awareness or training or conversation* or engag* or promotion* or story or stories or experienc* or sharing or educator* or education* or educating or encourag* or teaching or counseling or information or knowledge or communicat*).ab,ti.) and (exp interviews/ or exp. questionnaires/ or (qualitative or ethnograph* or phenomenol* or grounded theor* or purposive sampl* or hermeneutic* or heuristic* or semiotics or lived experience* or narrat* or life experience* or cluster sample* or action research or observational method* or content analys* or thematic analys* or constant comparative method* or field stud* or theoretical sampl* or discourse analys* or focus group* or ethnological research or ethnomethodolog* or interview* or mixed method* or mixed model* or mixed design* or survey* or questionnaire* or anecdote*).ab,ti.)	Results = 148

## Appendix 2

**Table 5:** Summary of critical appraisal of study quality

Domain	Berry & Planalp	Brighton et al.	Clarke et al.	Foster	Jones et al.	Planalp & Trost	Planalp et al.	Rocque et al.	Seymour et al.
Study purpose: Was purpose or research question stated?	Yes	Yes	Yes	Yes	Yes	Yes	Yes	Yes	Yes
Literature: Was relevant literature reviewed?	Yes	Yes	Yes	Yes	Yes	Yes	Yes	Yes	Yes
Study design: Was a theoretical perspective identified?	Yes	Yes	Yes	Yes	No	Yes	No	Yes	Yes
Sampling: Was the process of purposeful selection described?	No	Yes	Yes	No	Yes	No	No	No	Yes
Was sampling done until redundancy in data was reached?	N.A.	Yes	N.A.	N.A.	N.A.	N.A.	N.A.	N.A.	N.A.
Was informed consent obtained?	Yes	Yes	No	No	Yes	N.A.	N.A.	Yes	N.A.
Data Collection: Was procedural rigor used?	Yes	Yes	N.A.	No	N.A.	Yes	Yes	Yes	Yes
Descriptive clarity: Complete description of site	No	Yes	No	No	Yes	No	No	Yes	Yes
Descriptive clarity: Complete description of participant	Yes	Yes	No	No	Yes	Yes	No	Yes	Yes
Description of role of researcher and relationship with participants	No	Yes	Yes	Yes	No	No	No	Yes	Yes
Identification of assumption and biases of researcher	No	Yes	No	Yes	No	No	No	Yes	No
Analytical rigor: Were data analyses inductive?	No	Yes	N.A.	Yes	Yes	Yes	Yes	Yes	N.A.
Were findings consistent with and reflective of data?	Yes	Yes	Yes	Yes	Yes	Yes	Yes	Yes	Yes
Auditability: Was a decision trail developed?	N.A.	Yes	N.A.	Yes	No	N.A.	Yes	N.A.	No
Was the process of analyzing the data described adequately?	Yes	Yes	No	N.A.	No	Yes	Yes	Yes	Yes
Theoretical connections: Did a meaningful picture emerge?	No	No	Yes	Yes	No	Yes	No	Yes	Yes
Credibility: Do descriptions and interpretations of participants capture the phenomenon?	Yes	Yes	Yes	Yes	No	No	Yes	Yes	Yes
Transferability: Can the findings be transferred to other situations?	Yes	Yes	No	No	No	Yes	Yes	Yes	Yes
Dependability: Was there consistency between data and findings?	No	Yes	Yes	No	No	Yes	Yes	Yes	Yes
Confirmability: Were strategies employed to minimize bias?	No	Yes	Yes	No	No	No	No	Yes	No
Conclusions: Were conclusions appropriate given study findings?	Yes	Yes	Yes	Yes	Yes	Yes	Yes	Yes	Yes
Implications: Were findings meaningful to “laypersons” and communication?	Yes	Yes	No	Yes	Yes	Yes	Yes	Yes	Yes
Total score:	60%	95%	61%	60%	50%	68%	60%	95%	84%

Quality appraisal results and scoring of included studies using McMaster University tool

## Abbreviations

UK: United Kingdom
US: United States
